# SEISMONOISY: A Quasi-Real-Time Seismic Noise Network Monitoring System

**DOI:** 10.3390/s24113474

**Published:** 2024-05-28

**Authors:** Giuseppe Ruzza, Rocco Cogliano, Ciriaco D’Ambrosio, Luigi Falco, Vincenzo Cardinale, Felice Minichiello, Antonino Memmolo, Angelo Castagnozzi, Giovanni De Luca, Annamaria Vicari

**Affiliations:** Istituto Nazionale di Geofisica e Vulcanologia, Sezione Irpinia, 83035 Grottaminarda, Italy; rocco.cogliano@ingv.it (R.C.); ciriaco.dambrosio@ingv.it (C.D.); luigi.falco@ingv.it (L.F.); vincenzo.cardinale@ingv.it (V.C.); felice.minichiello@ingv.it (F.M.); antonino.memmolo@ingv.it (A.M.); angelo.castagnozzi@ingv.it (A.C.); giovanni.deluca@ingv.it (G.D.L.); annamaria.vicari@ingv.it (A.V.)

**Keywords:** seismology, real time monitoring, seismic noise, background seismic noise level, seismic noise trend

## Abstract

This paper introduces SEISMONOISY, an application designed for monitoring the spatiotemporal characteristic and variability of the seismic noise of an entire seismic network with a quasi-real-time monitoring approach. Actually, we have applied the developed system to monitor 12 seismic networks distributed throughout the Italian territory. These networks include the Rete Sismica Nazionale (RSN) as well as other regional networks with smaller coverage areas. Our noise monitoring system uses the methods of Spectral Power Density (PSD) and Probability Density Function (PDF) applied to 12 h long seismic traces in a 24 h cycle for each station, enabling the extrapolation of noise characteristics at seismic stations after a Seismic Noise Level Index (SNLI), which takes into account the global seismic noise model, is derived. The SNLI value can be used for different applications, including network performance evaluation, the identification of operational problems, site selection for new installations, and for scientific research applications (e.g., volcano monitoring, identification of active seismic sequences, etc.). Additionally, it aids in studying the main noise sources across different frequency bands and changes in the characteristics of background seismic noise over time.

## 1. Introduction

The basis of seismology and its research applications lies in the study of elastic wave propagation, the sources that generate these waves, and the structures through which they propagate. The main instrument used for measuring the intensity over time of the Earth’s vibration produced by different sources is the seismic station.

A seismic network consists of several seismic stations working together to collect and analyze data, making it an effective tool for monitoring ground motion in an area. The size and configuration of a seismic network is related to several factors, such as the extension of the area under observation and the location of potential seismic sources of strong motion (e.g., active faults, volcanoes, etc.). The data provided by a seismic network can be used for several purposes. In fact, it is the only system that can determine the location and magnitude of earthquakes at different scales, from global to national or local [1]. Other common purposes and applications of the data from seismic networks are the study of earthquake sources, research on the deep Earth’s structure, monitoring and studying volcanic activities, etc. [2,3,4].

The ground vibration measured on the Earth’s surface is the sum of several contributing sources with different motion characteristics both in terms of intensity and spectral frequency signature, which is why it is not possible to extrapolate and monitor a single source; therefore, the concept of noise must also be introduced in seismic study applications.

The definition of seismic noise in a signal recorded by a seismic station is not unique but varies according to the objective of each specific study. On the other hand, since most seismic networks are set up to locate seismic events such as earthquakes, volcanic activities, and other seismogenic sources, all other vibrations in the absence of transient signals from earthquakes are considered seismic background noise.

Seismic noise is caused by a variety of different, spatially distributed, mostly uncorrelated and often continuous sources. Although the source of seismic noise is not fully understood, we can divide the sources of seismic noise into two main categories. The first one is natural background noise, which may include the following: atmospheric pressure variation [4,5,6,7,8], temperature change [9], ocean waves [10,11,12,13,14], tide wind and cyclone [15,16,17], and the effects of wind on trees [18].

Seismic noise related to natural sources is characterized by a low frequency, typically less than 1 Hz [19,20].

In the second category, all seismic noise generated by anthropogenic sources is classified, characterized by frequencies higher than 1 Hz [19,20].

Another factor that differentiates natural seismic noise from anthropogenic noise is the variability of its intensity over time. In fact, noise linked to anthropic sources reveals a stronger variation between daytime and nighttime hours as well as between different days of the week [21]. This behavior is related to different human activities that change over time [22]. In contrast, low-frequency seismic noise exhibits less temporal variation in terms of level of noise.

Although the intensity of natural seismic noise can change considerably from site to site, the common procedure for evaluating the level of seismic noise was established by Peterson [23], who developed a global noise model that can be used as reference, where two level boundaries were defined: the new high-noise model (NHNM) and the new low-noise model (NLNM). These limits represent the upper and lower limit of background natural noise represented in the Power Spectral Densities (PSDs) of vertical true ground acceleration over the entire seismic frequency band.

Therefore, the NLNM and NHNM are the maximum and minimum levels of expected background seismic noise in seismic recordings made at the Earth’s surface, so they can be used as a comparison system to assess the level of seismic noise present at a site.

The level of seismic noise measured at a site is given by the sum of the natural background noise and by a contribution that comes from the installed instrumentation (i.e., from the sensor and digitizer used). The contribution of the noise of the seismic instrumentation, and thus its intensity and frequency contribution, is related to the characteristics of various mechanical and electronic parameters.

The main parameters that most influence the sensor noise are their bandwidth, dynamic range, and self-noise.

The bandwidth parameter is the frequency range in which the instrument has a flat response to ground speed, broadband, and very broadband seismic sensors; the most used sensors in seismic networks at global, national, and regional scales can cover more than four decades of logarithmic frequency.

The dynamic range expressed in decibels (dB) quantifies the ratio between the largest and smallest recoverable level. Broadband seismometers have a dynamic range typically around 160 dB. Such a wide dynamic range allows for vibration levels from very strong to very weak to be observed simultaneously without appreciable distortions.

Instead, the noise related to electronic sources in both a sensor and signal digitizer is made up of two main components: white noise and flicker noise, with the latter also being known as 1/f noise or pink noise.

The power spectral density of 1/f noise is inversely proportional to frequency, so this noise increases at the lowest frequency.

The definition of seismic noise, however, is not unambiguous in scientific research. In fact, while in the field of earthquake monitoring applications the least seismic noise possible is necessary, in order to improve the signal-to-noise ratio and therefore increase the ability to detect a seismic event, the vibrations induced by local seismic events of minor intensity or by teleseisms or teleseismic events can be hidden by the intensity of the noise, thus reducing the magnitude of completeness.

In other research applications, however, the background signal is not seen as noise; in fact, there are several research applications that use natural seismic noise as a useful signal. Examples of applications of the use of background noise are imaging and land monitoring by obtaining Green’s functions between pairs of stations from the stack of their noise cross-correlograms [24], indirect subsurface exploration through ambient noise tomography [25,26], determination of significant ocean wave heights [27], determination of the response of the seismic amplification of a site through the HVSR technique [28,29,30,31], study of the temporal and spatial changes in the property statistics of seismic noise as a precursor or preparation for strong earthquakes [32,33,34,35,36], characterization of ambient seismic noise for improving the design of long-term seismograph installations [37], and spectral characteristics of the continuous seismic noise signals to understand the dynamics of a hydrothermal source [38].

The use of environmental seismic noise, particularly its characteristics, its variations in space and time, and its potential sources, has been applied in several scientific studies aimed at elucidating its implications in relation to entire seismic networks at different scales, from global to national. For example, Dìaz et al. [39] studied the seismic background noise characteristics of the IberArray broadband seismic network; this seismic network is located in southern Iberia and northern Morocco. The authors, through the estimation of the PDF applied on 55 seismic stations, have identified the main sources of seismic noise in different frequency bands and studied their variations over time. This study was aimed at identifying operational problems in order to improve the quality of the network with the possible movement of some stations and/or changes to sensor isolation techniques.

Vassallo et al. [40] studied the environmental noise recorded by the Irpinia Seismic Network (ISNet) in order to evaluate the noise spectrum for each station as a function of time and the earthquake detection threshold of the network itself.

Krishna et al. [41] studied the characteristics of different types of noise using the PSD of seismic stations located along the Western Ghats (India). Poveda-Brossard et al. [42] analyzed the seismic noise of the broadband seismic network included in the Cuban National Seismic Service through the evaluation of the noise PSD in order to study the quality of the installation of seismic sensors and the other factors involved in the noise level of the entire seismic network.

Gaebler et al. [43] studied the detection capabilities of a minimal observable body wave magnitude of a seismic event based on the measurements of ambient noise levels at the global scale of the International Monitoring System (IMS). Uthaman et al. [44] provided, with an analysis of PSD, a detailed characterization of the ambient noise of the Sikkim Himalaya area and the Himalayan foreland basin to evaluate the performance of 27 new broadband seismic station installations.

Fornasari et al. [45] have determined with their research the characteristics of the background noise of the Italian strong-motion network on the basis of continuous data collected in 2022. This study analyzes the spatial and temporal characteristics of the background noise calculated through the PSD.

D’Alessandro et al. [46] used data from 233 broadband seismic stations to evaluate the spectral characteristics of seismic noise at sites of the Italian Seismic Network and their spatiotemporal variability based on 4 years of data.

In this article, we want to present a SEISMic nOise Network mOnItoring System, “SEISMONOISY”; this is a tool that allows us to continuously monitor in quasi-real time the noise level of a seismic network. This developed tool, therefore, allows us to monitor and study both the quality and performance of a seismic network under observation and also the behavior and variation in background noise over time.

SEISMONOISY is capable of analyzing a large number of seismic sensors, enabling it to monitor entire seismic networks with national coverage. As of the date of this publication, the developed system monitors approximately 427 seismic stations. This number may change over time due to new installations or being decommissioned.

The seismic stations belong to different seismic networks installed within the Italian territory, of which approximately 76% of the monitored seismic sensors belong to the main seismic network present in Italy, namely the Italian National Seismic Network “IV”(Rete Sismica Nazionale “RSN”), managed by the Istituto Nazionale di Geofisica e Vulcanologia “INGV”.

The remaining monitored stations belong to smaller regional seismic networks, the use of which allowed us to increase the number of measurement sites in order to achieve optimal station density.

**Figure 1:**
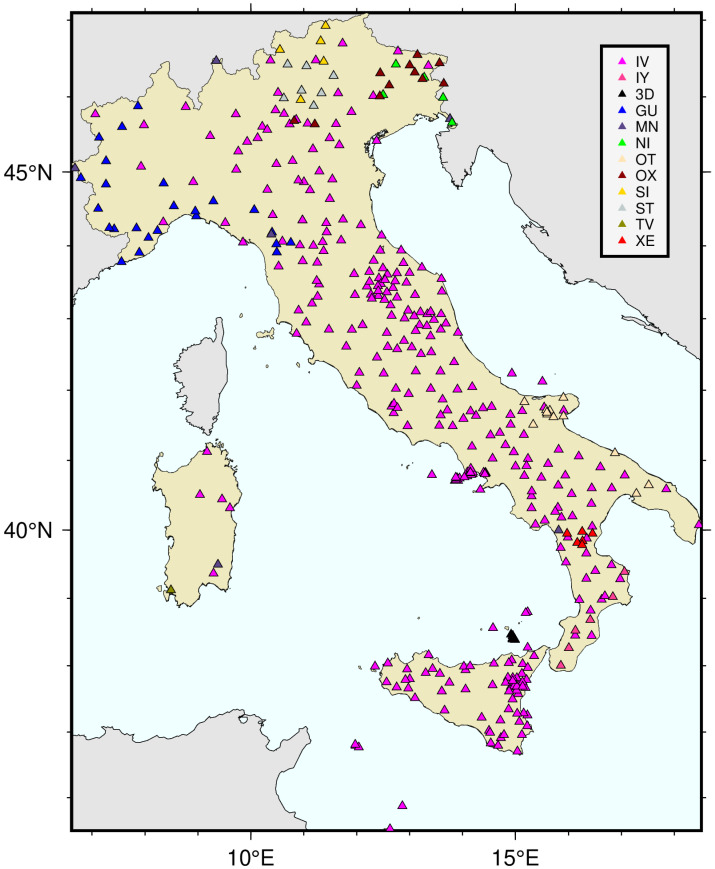
Location map of the different seismic networks and their seismic sensors, taken into account by SEISMONOISY.

The regional seismic networks taken into consideration are the Broadband Seismic Network of North Eastern Italy “NI”, the Regional Seismic Network of North Western Italy “GU”, the Broadband Seismic Network of the Mediterranean “MN”, the Rete Sismica Unical “IY”, OTRIONS seismic network “OT”, the Seismic Network of North East Italy “OX”, the INGV Experimental Network “TV”, the Pollino-Near Fault Observatories “3D”, the INGV temporary network for monitoring the island of Vulcano “3D”, the Trentino Seismic Network “ST”, and the Seismic Network of the Province Südtirol “SI”. Figure 1 shows a map of the position of all the stations monitored by our system divided by seismic networks.

SEISMONOISY uses the calculation of the Probability Density Function (PDF) based on the calculation of noise Power Spectral Density (PSD) proposed by McNamara and Buland [47] to analyze and characterize the noise of individual monitored stations. This method has become increasingly common in numerous environmental noise studies [40,48,49,50] in which the energy distribution of ambient noise using PDF is compared to the global ambient noise proposed by Peterson [23].

Then, our software also compares a noise level analysis through the PDF function with the minimum and maximum NLNM and NHNM levels. From this comparison, a dimensionless seismic noise value called the Seismic Noise Level Index (SNLI) is extrapolated.

The calculation of site noise is made on the basis of velocity signals and not acceleration signals; this choice arose from the fact that the signal of velocimeter sensors has such a response that they are better suited to record ambient noise within the frequency band of interest. The seismic noise data obtained in quasi-real time expressed in the SNLI value are useful for characterizing the performance of the seismic stations and therefore of the entire seismic network, also allowing for the detection of operational problems in relation to possible faults or malfunctions of the installed instrumentations.

Instead, through historical seismic noise data, it is possible to study its characteristics and its variation over time. This can be useful, for example, in investigating and evaluating new sites for future installations, or even evaluating possible changes in the locations of existing seismic stations to optimize the network performance. In addition to the assessments that can be made on individual seismic stations based on its noise level, as previously mentioned, the quasi-real time calculation of seismic noise performed by SEISMONOISY across an entire seismic network enables evaluations on a larger scale, from regional to national.

At this wider scale, observations and studies can be conducted not only on the quality and functionality of single seismic stations but also on phenomena related to seismic activity, such as those in seismogenic areas.

For instance, it is possible to observe and monitor over time, and thus study, the behavior and seismic activity patterns in active faults and volcanic areas.

Furthermore, the natural seismic background level reported by the system can be connected to other types of processes, not only generated at the local or national scale but also at the global level (e.g., atmospheric phenomena and the strength of ocean microseisms). These factors can affect the level of background noise across the entire seismic network on a national scale, even if the sources are external and distant.

Finally, the SEISMONOISY system, as designed, can be easily applied also to other networks besides the Italian ones. This capability allows our system to potentially expand the observation area and enable the study of phenomena that induce seismic vibrations on an increasingly larger scale and under different geological and topographical conditions.

## 2. Data

The seismic data recorded by the velocimetric sensors used by our system are gathered in a common database, which can be requested and downloaded via the Internet. In particular, the seismic data are requested and retrieved through the Orfeus European Integrated Data Archive (EIDA) [51,52] federation and International Federation of Digital Seismograph Network (FDSN) standard protocols under an open definition compliant license (https://www.fdsn.org/datacenters/detail/INGV/) (accessed on 23 May 2024).

The calculation of seismic noise occurs by analyzing 12 h long data windows. This allows for the division of the day into two parts. Specifically, we chose to analyze seismic data from 8:00 a.m. to 8:00 p.m. and from 8:00 p.m. to 8:00 a.m. This temporal scheme was chosen to attempt to separate the daytime component from the contribution of seismic noise during the nighttime. This enables us to isolate the noise primarily originating from anthropic sources, whose greatest contribution typically occurs during daylight hours.

For this reason, the software has been programmed to automatically download and subsequently process the data of all stations every 12 h. So, for example, the noise level reported by our software at 8:00 a.m. is the result of the noise recorded in the seismic trace of the previous 12 h, and so on.

Below is a brief description of both the permanent and temporary seismic networks to which all the seismic sensors analyzed belong.

- IV: Italian National Seismic Network [53]. This network is the nationwide permanent seismological network operated by the Istituto Nazionale di Geofisica e Vulcanologia ‘INGV’, consists of various types of seismic sensors (i.e., short period, broadband, and accelerometric instruments) which are located at many sites, and it is the largest Italian seismological network by a number of sensors and to a geographical extent.
- NI: North-East Italy Broadband Network, https://rts.crs.inogs.it (accessed on 23 May 2024). This network consists of 43 stations, of which 24 are broadband, 15 are short period, and 4 are intermediate period. The network is managed by OGS also on behalf of the Friuli Venezia Giulia and Veneto Regions. The network also provides data to the national seismic surveillance system, with real-time data exchange with the Civil Protection Department and the National Institute of Geophysics and Volcanology (INGV).
- GU: Regional Seismic Network of Northwestern Italy [54]. The Regional Seismic Network of Northwestern Italy (abbreviation: RSNI; international network code: GU) is currently composed of 32 seismic stations directly managed by the University of Genoa (Distav) located throughout the northwestern part of the Italian peninsula.
- MN: Mediterranean Very Broadband Seismographic Network [55]. The Mediterranean Very Broadband Seismographic Network (MedNet) constitutes a backbone network of highest-quality broadband stations in the countries surrounding the Mediterranean Sea. The MedNet project, which operates and maintained the network, is led by the National Institute for Geophysics and Volcanology (Italian: Istituto Nazionale di Geofisica e Vulcanologia, INGV), but it receives significant contributions from many cooperating geophysical institutes from the countries involved. All stations transmit sensor data in real time to the INGV data center in Rome (Italy).
- IY: Rete Sismica Unical [56]. This seismic network is devoted to monitoring the earthquake events in the Calabria Region of Italy for experimental purposes; more information can be retrieved in http://www.sismocal.org (accessed on 23 May 2024).
- OT: OTRIONS seismic network [57,58]. This local seismic network deployed in the Apulia area (southern Italy) consists of 12 seismic stations equipped with short-period velocimeters connected in real time to two seismic rooms in Bari and Taranto (Puglia province, Italy). The data, also regarding micro seismicity, are used to produce both seismic and attenuation tomography. The network is connected to the INGV (National Institute of Geophysics and Volcanology) for Civil Protection purposes.
- OX: North-East Italy Seismic Network [59]. The North-East Italy Seismic Network (Rete Sismica dell’Italia Nord-Orientale) is a permanent seismological network operated by the National Institute of Oceanography and Applied Geophysics—OGS (Istituto Nazionale di Oceanografia e di Geofisica Sperimentale ‘OGS’). Originally registered under the FV and NI network codes, it was unified in 2016 into the single OX network code. The network currently consists of 22 broadband and 19 short-period seismic stations; all data are acquired at the Seismological Research Centre, OGS, in Udine.
- ST: Trentino Seismic Network [60]. This is a regional short-period and broadband seismic network operated by the Geological Survey (Civil Protection Department) of the Autonomous Province of Trento (Northeastern Italy); this network is used for monitoring and civil defense purposes.
- XE: POLLINO-Near Fault Observatories [61]. This network consists of 10 semi-permanent broadband seismic stations; the network began operations in spring–summer 2022.
- 3D: INGV temporary network for seismic monitoring of the island of Vulcano [62]. The Etneo Observatory of Catania of the INGV has installed a temporary network to improve the seismic monitoring of the volcanic island after a significant change in various volcanic parameters.
- SI: Province Südtirol. More information can be retrieved from https://terremoti.ingv.it/en/instruments/network/SI (accessed on 23 May 2024).
- TV: INGV experiments network. More information can be retrieved from https://terremoti.ingv.it/en/instruments/network/TV (accessed on 23 May 2024).

As reported in Table 1, there is large variety in the sensors installed; there are ones with different characteristics, among which the main is the bandwidth, and there are short-period, broadband, and very broadband sensors. We have not considered the division and analysis of sensors according to their type, as the main purpose of SEISMONOISY is to monitor the status and quality of the entire network regardless of the type of sensor throughout the frequency band of seismological interest.

In addition to the seismic data, the corresponding metadata in XML format [63] are also downloaded for each seismic station. Metadata are crucial points of information for the noise processing steps of the seismic traces.

Furthermore, metadata files are downloaded every 12 h so that the software always receives the most up-to-date information on the type and characteristics of the seismic station’s instrumentation configuration of both the sensor and the digitizer, because these components may have any changes due to upgrades or replacements due to repairs.

## 3. Methods

As reported in the Introduction, we chose seismic noise as the main technique to analyze, and the calculation of the PSD function of the seismic signal recorded by the stations, but since the noise level changes over time, we carried out the statistical analysis proposed by McNamara and Buland [47], where the PSD curves were used to define a Probability Density Function (PDF) of the seismic noise. Finally, from the PDF data, we can obtain the final representative PSD of the seismic station under observation.

In particular, we have implemented PSD spectral analysis because it is more appropriate than other spectral analyses for quantifying the strength of seismic noise, which is a stationary stochastic process without a defined phase spectrum, thus characterized by infinite energy but finite power, unlike transient signals [64]. For these reasons, PSD is a powerful tool for evaluating the long-term performance of a seismic station by providing an overview of the cumulative spectral content over time. This approach allows for the examination of all noise sources related to a site (e.g., the artifacts related to station operation, the self noise of the station, the cultural noise, the seasonal meteorological changes, and other natural or artificial factors). The sum of all these elements, and the variation in their contributions over time, constitute the background seismic noise level characteristic of a given site.

SEISMONOISY was developed on the Python platform, which offers the user all the features of a complete programming language, including a large collection of open-source scientific modules, and is one of the most popular programming languages even in scientific applications [65].

We chose Python because it is an open-source platform and there are no licensing restrictions compared to other programming languages used in scientific applications, such as MATLAB (http://www.mathworks.com) (accessed on 23 May 2024).

As the main python toolbox for processing the seismological data, we have adopted ObsPy (http://www.obspy.org) (accessed on 23 May 2024) [66].

This library allows users to read and process seismic data and metadata in the different formats commonly used in seismological applications (e.g., SEED, .XML, etc.). ObsPy is successfully used in the scientific community as a tool for processing seismological data [67,68].

**Figure 2:**
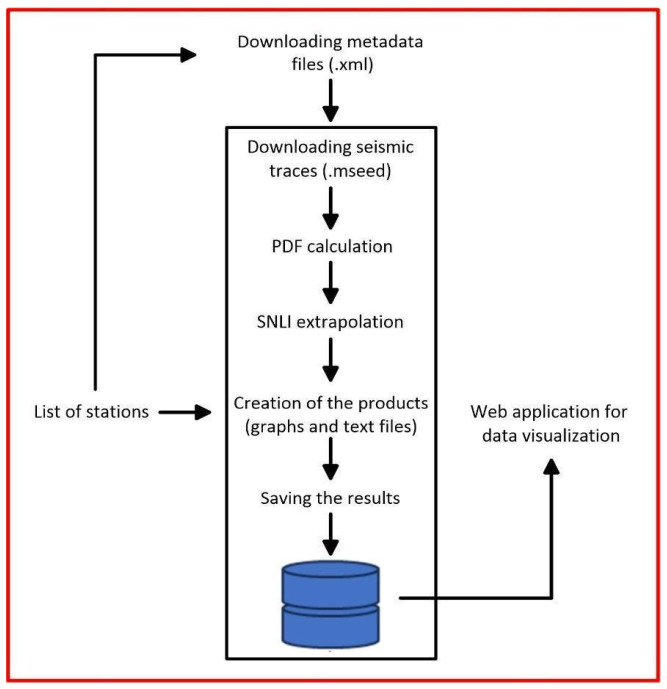
Simplified flow diagram of SEISMONOISY application.

Figure 2 shows the flow diagram of the processes performed by the SEISMONOISY application. As reported before, SEISMONOISY is a Python script and can be run on both the Linux and Windows environments through a command line.

## 4. SEISMONOISY Application

**Figure 3:**
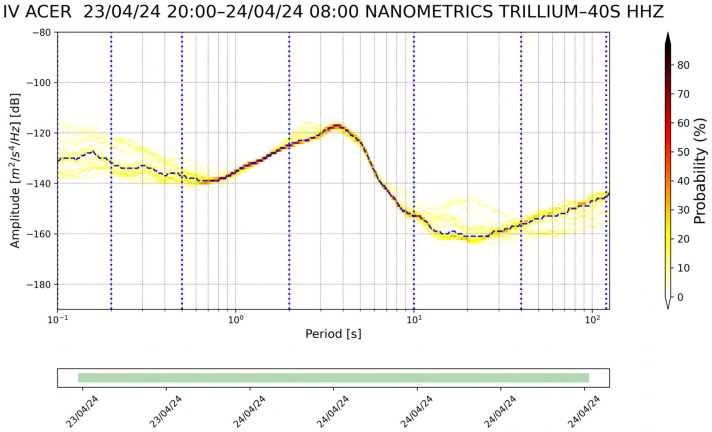
Example of result of the PDF function for the station ‘ACER’ belonging to the IV seismic network for the Z component. The blue dash line represents the PSD equal to 50% percentile.

**Figure 4:**
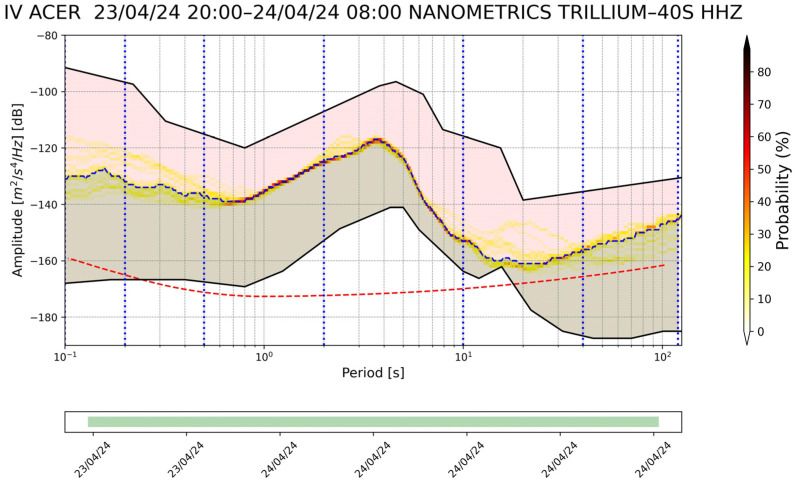
Example of final graph for Z component produced after calculation of the PDF and SNLI. The two black lines, the upper and lower ones, represent the NHNM and NLNM of Peterson’s (1993) curves respectively; the red fill area represents the area between the 50% percentile PDF curve and the NHNM, and the green fill curve represents the area between the 50% percentile PDF curve and the NHNM. The figure also shows the period intervals used to derive the different SNLI values, and the dashed red curve represents noise value characteristic of the seismic sensor installed on the seismic station declared by the manufacturer. Lastly, the blue dashed line represents the PSD value equal to the 50% percentile of the PDF function.

**Figure 5:**
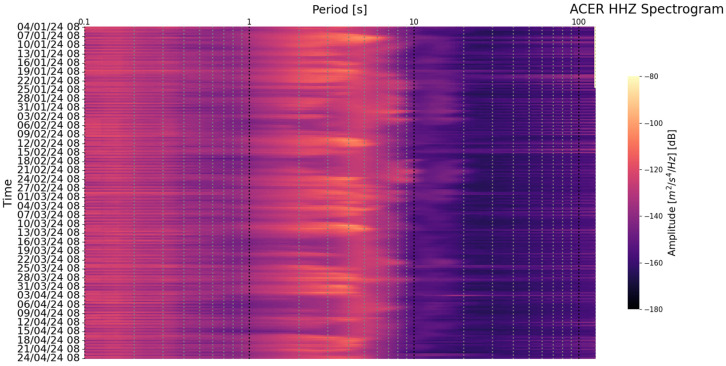
Example of the 50% percentile plot of the PDF function over time in a spectrogram format display for the Z component of station ‘ACER’.

**Figure 6:**
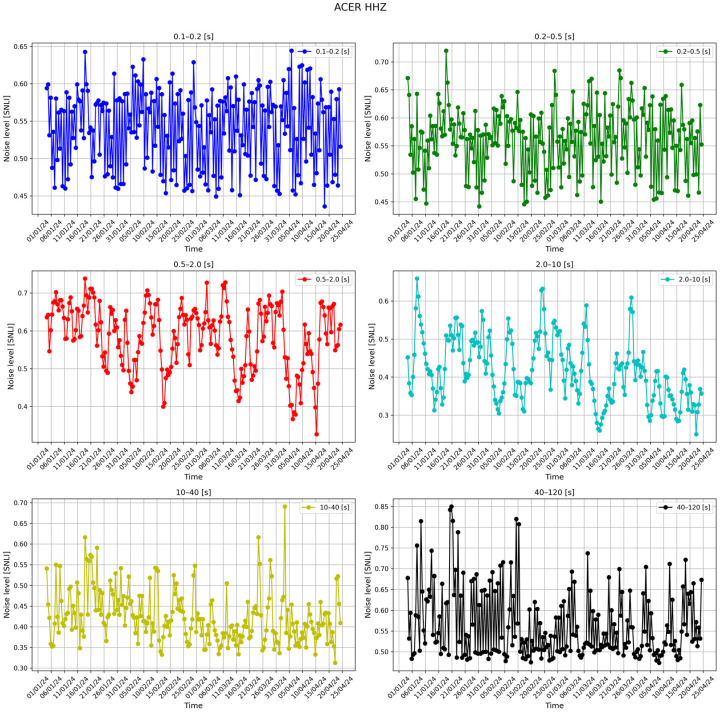
Graphs of the trend of the SNLI value for each period interval for the Z component of the ‘ACER’ station.

**Figure 7:**
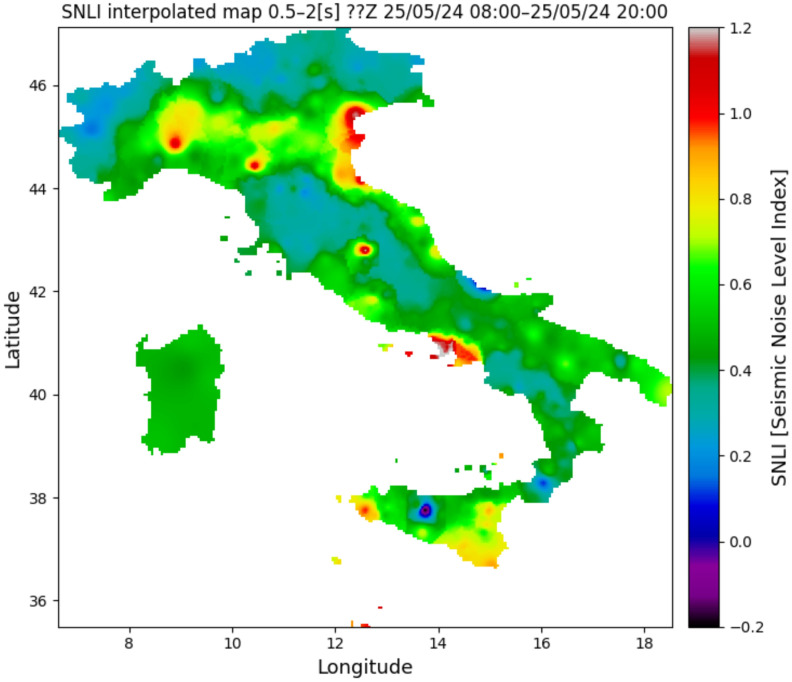
Example of the interpolation map of the SNLI value for the interval period 0.5–2 s for the Z component. Since the stations of the seismic networks considered by SEISMONISY are heterogeneous, they are identified with different channel codes. Specifically, the first two letters refer to the properties of the sensor, and the third letter to the channel orientation. For example, HH stands for High Broad Band and High Gain Seismometer sensors, and EH stands for Extremely Short Period and High Gain Seismometer sensors. Since the interpolation map is produced based on both types of sensors, the title of the image indicates ??Z because the interpolation is done with both types of sensors and only the direction is uniquely identified.

As can be seen from Figure 2, there are different steps; each one performs a different task, and below is a description of each one:

1. Station list is the main text file in which the information of the seismic stations that will be considered in the monitoring process is saved. This file contains the following information for each station: the network code and the code of the corresponding station (e.g., IV, ACER). The use of a simple text list on which all subsequent processes are based facilitates the insertion or deletion of seismic stations.
2. The first step of the whole process is to download the metadata of each station in the station file list. The metadata in station .XML format are downloaded from INGV’s webservice https://webservices.ingv.it/fdsnws/station/1/ (accessed on 23 May 2024). The download of these data is performed every 12 h in order to obtain always the most updated information of seismic stations.
  Based on the downloaded metadata files, a text file is created in which the main information of the individual station is saved (i.e., net code, station code, type of sensor, latitude and longitude, etc.); this file is generated every 12 h.
3. After downloading the metadata files, the process involving the seismic traces starts. First of all, the seismic trace of each seismic component is downloaded from the INGV webservice https://webservices.ingv.it/fdsnws/dataselect/1/ (accessed on 23 May 2024) in miniSEED format. For each seismic component, the PDF is calculated through the PPSD class of ObsPy based on the approach of McNamara and Buland [47].
  Before calculating the PDF function, the different PSD curves are calculated over a period interval between 0.1 and 120 s. The calculation involves dividing the 12 h seismic trace into 60 min windows, with these overlapping by 50%, each subsequently divided into 15 min subwindows with 75% overlap. For each subwindow, the average and long-term trend are removed, tapering is applied with a 10% sine function, and transformation is performed with the FFT algorithm. Finally, the subwindow segment is averaged to provide a PSD every 60 min. The instrument response is then removed from the PSD. This last process also converts the PSD from ground velocity to ground acceleration. The PSDs are calculated in decibels with respect to acceleration of 1(m/s^2^)^2^/Hz in order to compare the final results with Peterson’s (1993) noise models. Transient signals, consisting also of earthquakes, are not removed from the seismic traces since they are low-probability occurrences with respect to ambient seismic noise. For each channel, the PDF is derived by overlaying the individual PSDs calculated previously. The parameters for constructing the PDF are a binning period of 1/3 of an octave and a smoothing period of 1/32 of an octave. A binning period of 1/3 of an octave can be a reasonable trade-off between the obtained spectral resolution and in the accuracy in the broadband noise source. Each raw frequency distribution bin is then normalized by the total number of PSDs to construct the PDF. A final approximate representative PSD of the whole twelve of our traces is extrapolated for the superimpose 50% percentile level of the PDFs. Finally, a graph of the results of this step is generated (Figure 3).
4. The fourth step involves the calculation of the Seismic Noise Level Index (SNLI). This is a dimensionless index that takes into account the maximum and minimum values of the Peterson (1993) curves. The noise level is calculated for different period intervals, so it is possible to study and monitor the contribution of the noise level throughout the range of interest. We divided the entire range of interest ranging from 0.1 to 120 s into a total of six ranges, namely 0.1–0.2, 0.2–0.5, 0.5–2, 2–10, 10–40, and 40–120 s. The calculation of the noise level index is performed in three steps: the first phase involves calculating the area subtended by the NHNM and NLNM curves of Peterson (1993) within the chosen interval. In the second phase, the area subtended by the 50% percentile PSD curve of the calculated PDF function and the NLNM curve is calculated in the same interval in which one is working. The last phase is the application of the division function between the value of the first area (i.e., the calculated one between the NLNM and NHNM curve) with the second area (i.e., area calculated between the 50% percentile curve and the NLNM curve). With this method, the calculated dimensionless index defined as SNLI represents the intensity of the noise in a period interval considered in relation to Peterson’s curves. The SNLI is a very simple value to immediately understand the state of the noise level in a certain period interval. In fact, an SNLI with a value between 0 and 1 indicates that the noise level is between the NHNM and NLNM curves, a value greater than 1 shows a noise level higher than the NHNM, and a lower value than 0 is related to a noise level lower than the NLNM. This index allows for, for example, in those cases in which the value is much lower than 0 or much greater than 1 an anomaly to be highlighted which can be correlated to a malfunction of the instrumentation or to a real state of the anomalous noise level. In Figure 4, the final figure generated is representative of the noise of a seismic station.
5. Product creation step. The main products generated by SEISMONOISY are essentially of two types: graphics and text files. There are four main graphs, the first one is the result of the PDF function (Figure 4), the second one is a spectrogram of the representative PDS equal to 50% of the percentile of the PDF function (Figure 5), the third graph concerns the trend of the SNLI values for all period intervals calculated (Figure 6) (this graph allows to monitor the noise level over time), and finally, the last figure represents the interpolation map of the SNLI value on the entire monitored seismic network (Figure 7). In particular, the interpolation map is generated using the pyidw python library [69] (https://github.com/yahyatamim/pyidw?tab=readme-ov-file) (accessed on 23 May 2024). This library is based on the Inverse Distance Weighting (IDW) geospatial interpolation method [70]. There are two main text files: in the first one, the numerical values of the PDS curve corresponding to the 50% percentile of the PDF function are saved, and in the second one, the SNLI values calculated for each period interval are saved.
6. The procedure described in steps 3–4–5 is carried out for all the stations present in the ‘list stations’ file described in step 1.
7. All textual and graphical products are saved within a repository, and the SEISMONOISY application will wait for the start of the next schedule to redo the entire procedure for the next data cycle.

The SEISMONOISY Python processing application runs on a virtual machine based on the Debian 12 operative system provided by two CPU cores and a total amount of 4 GB of RAM. With this configuration, the processing time to complete the seismic noise analysis for all the monitored stations is about 4 h.

## 5. Web Application for Data Visualization

To consult the results processed and produced by SEISMONOISY, a dedicated web application has been developed (https://seismonoisy.gm.ingv.it/) (accessed on 23 May 2024). The application is a single-page application (SPA) [71], that is, a web application designed to run entirely on a single web page, and unlike a traditional multiple-page application (MPA) which requires reloading the page every time the user navigates between different sections or performs actions, an SPA dynamically loads the necessary content and updates only the relevant parts of the page without reloading the entire page. The SPA web application approach was chosen because it provides a seamless user experience as the application responds quickly to user actions without having to continuously load all resources.

The web application was developed entirely using the JavaScript programming language [72]. To enhance the user experience, AJAX [73] was implemented, enabling asynchronous data loading and decoupling it from the GUI (Graphical User Interface) loading. For dynamically updating the page content, the open-source framework Angular [74] was utilized, allowing for code execution directly in the browser without continuous server queries.

**Figure 8:**
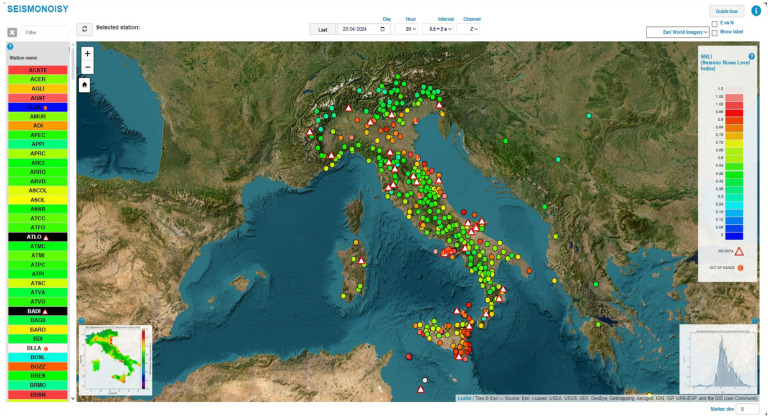
Main view of the web users’ interface; this web application allows the users to consult the products generated by the SEISMONOISY application.

The main window of the web interface (Figure 8) shows the map in which all the monitoring seismic stations are georeferenced, represented by a circle spot symbol. The color of the symbols connected to seismic stations is determined by their SNLI value. The SNLI value displayed for each seismic station is associated with the selected time period interval and seismic component from the dropdown menus available in the interface.

**Figure 9:**
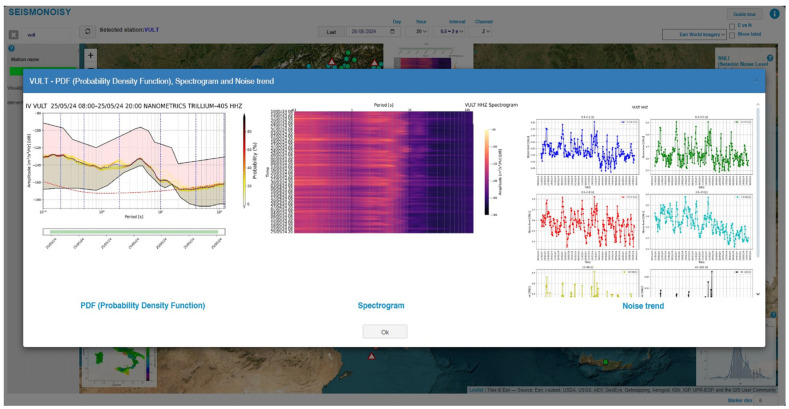
Example of the pop-up window opened after clicking on a station, in this case ‘VULT’.

In the upper right position, the color scale used for representing the SNLI value is shown. In the left position, there is the list of all the seismic stations. Also, in this list, the color is related to the SNLI, and the same color scale reported on the right is used. In the main central window in the left bottom position, the interpolation map of the SNLI value is shown, and in the right below position, the statistical distribution of the SNLI value for all the stations is shown. Also, the interpolation map and the statistical distribution graph are updating with respect to the temporal parameter chosen in the drop-down menus. By clicking on the station symbol in the main window or on the name of a station listed on the left, a pop-up window opens, allowing for the visualization of the PDF, spectrogram, and the SNLI noise trend graphs for the selected site (Figure 9).

**Figure 10:**
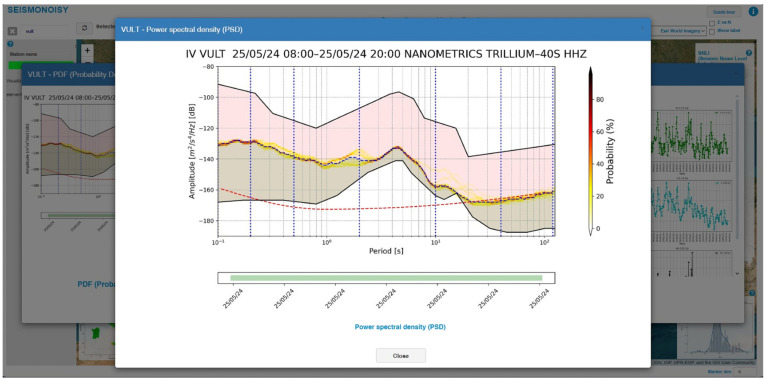
Example of detail of the PDF graph of the station ‘VULT’.

After opening the pop-up window of the chosen station (Figure 9), all the graphs shown in the pop-up window can be visualized, with more details revealed by clicking on the image of interest (Figure 10).

**Figure 11:**
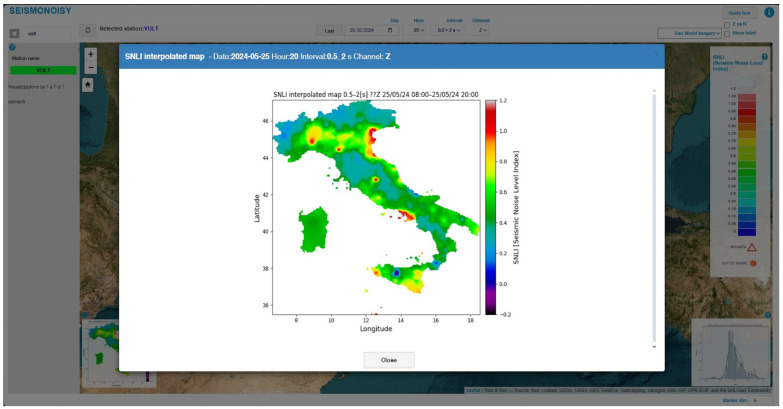
Example of the pop-up window that shows the detailed SNLI interpolation map. The color scale used for this map does not correspond with the range value of the color scale used in the main window (Figure 8), but it is calculated based on the maximum and minimum value of the SNLI value used for interpolation.

Also, the interpolation map and the SNLI statistical distribution map can be visualized; more details can be acquired by clicking on the corresponding miniature image present in the main window (Figure 11).

## 6. Results

As introduced in the previous chapters, SEISMONOISY was developed for monitoring the noise level of a seismic network. The primary objective of the software is undoubtedly to oversee the operational status and performance of the network, as well as individual seismic stations. The secondary goal is to monitor the distribution and trends of noise across the network; this can be useful for scientific purposes or for monitoring local events (e.g., volcanic activities, probable seismic sequences in seismogenic areas, etc.). Below, some examples of observations that can be made through the developed monitoring tool are reported.

### 6.1. Status of the Single Seismic Station

An SNLI value too high or too low measured at a seismic station might indicate anomalies or malfunctions of a seismic sensor; these come reported with a specific warning sign that indicates an SNLI value is out of the range of the color scale in current use.

A first clue in the localization of seismic stations with anomalous behavior can be accomplished through the observation of the SNLI; in fact, it might be a value out of range, or a station may have an anomalous value indicated by quite a different color scale from nearby stations.

**Figure 12:**
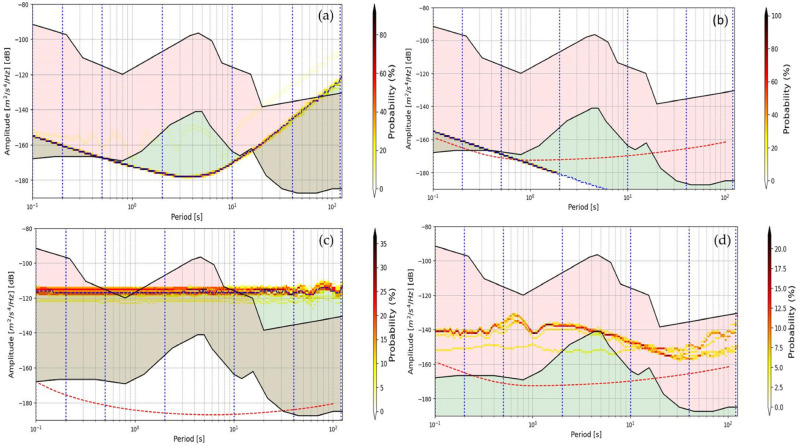
The image shows four example PDF function graphs from four different seismic stations (**a**–**d**). Each graph highlights potential malfunctions that may be caused by issues of the sensor, the digitizer, or both.

**Figure 13:**
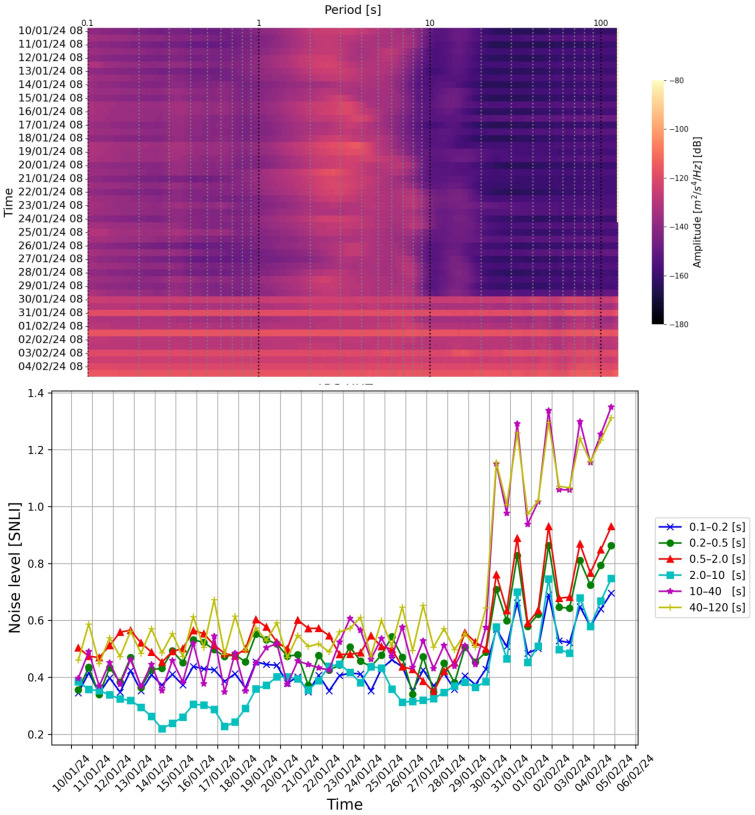
Example of the spectrogram and the graph of the SNLI trend of station (c) reported in Figure 12. From the observation of these two graphs, it is possible to understand when the station began to exhibit anomalous behavior linked to probable malfunctions. For this station, the anomalous behavior started from 30 January 2024.

From the first control of the PDF graph, it is possible to verify if there is a real anomaly related to instrumentation response or is due exclusively to environmental noise behavior. In Figure 12, some examples of PDF graphs are reported, in which it is possible to observe anomalies of noise behavior of four seismic stations; this can be due to malfunctions of the seismic station instrumentation. By observing the spectrogram and the graph of the trend of the SNLI value, it is possible to understand whether it is a temporary problem and evaluate when it started to occur (Figure 13).

### 6.2. Performance of the Seismic Network

The noise level of a seismic station is the sum of the natural background seismic noise level and the mechanical and electronic instrumental noise, so the total noise level defines a lower limit for the ability to detect and characterize various seismic signals of interest. The background noise level is a systematic bias in arrival times because the amplitude of the seismic phase must arise above the station noise level [75]. The contribution of the intrinsic noise of the sensor becomes more accentuated at low frequencies and is therefore mainly related to corner frequencies of its amplitude frequency response. For example, broadband sensors (e.g., >40 s) will present a low noise contribution at low frequencies, contrary to narrower band sensors.

**Figure 14:**
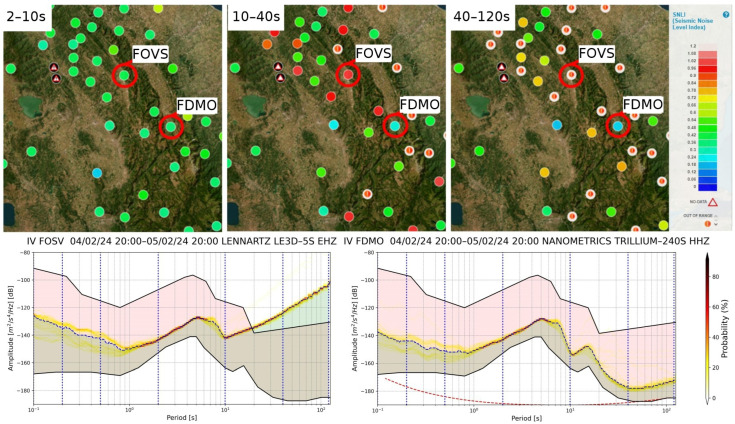
Examples of different noise contributions related to the different bandwidth of the sensors. In particular, the sensor of the ‘FOSV’ station characterized by a period of 5 s shows an increasingly greater contribution to the noise level at lower frequencies, while the broadband sensors installed at the ‘FDMO’ station show the smallest variation in the noise level in the different period bands.

Since the SEISMONOISY computes the SNLI for different range periods, it is possible, especially at lower period ranges (i.e., 10–40 s and 40–120 s), to observe a different self-noise contribution due to different kinds of sensors (Figure 14). The observation of site noise, therefore, is not only linked to natural background noise but also to instrumental noise, and it can be useful for understanding and consequently planning if it is deemed necessary to improve the performance of the network in certain areas.

### 6.3. Scientific Purposes Applications

Although the main objective of the developed software is to monitor the noise level of the seismic network in order to recognize possible problems related to the individual seismic station, it is also suitable to improve the performance of the seismic network as described in the previous sections. The noise level can also be used for scientific observation purposes and the monitoring of natural seismogenic phenomena (e.g., active volcanic area, analysis of seismic sequences, etc.).

**Figure 15:**
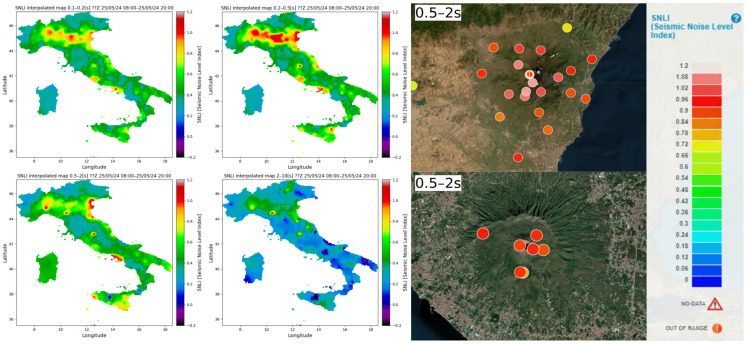
In the left example of interpolation maps of the seismic level noise expressed in SNLI, on the right, the noise level is measured in the areas of active volcanic context.

In Figure 15, some examples of possible observations based on the distribution of the noise level are depicted. Particularly, in the figures of the noise interpolation map presented on the left side of Figure 15, it is possible to observe and study the distribution of the noise in different period intervals.

**Figure 16:**
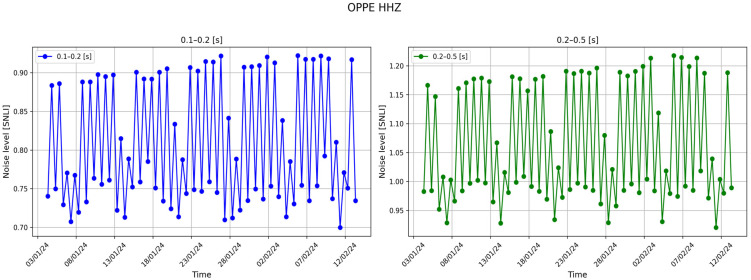
Trend of the noise level registered at the ‘OPPE’ station. This station is located in the Po plain in Oppeano town (Verona Province, Italy).

For instance, it is noticeable how, in Italy, the noise distribution varies across different period bands. For example, the areas characterized by the highest noise levels in the 0.1–0.2 s and 0.2–0.5 s bands are predominantly located in northern Italy, especially in the Po plain area situated between the Alps and the Apennines. In Figure 16, the noise levels of the aforementioned bands are depicted, demonstrating a strong correlation between noise trends and anthropogenic activities. Specifically, higher noise levels are evident between Monday and Friday, whereas lower noise levels are observed on Saturdays and Sundays (Figure 16).

But the high noise level of the Po plain may also be due to the lithology of this area; in fact, this area is one of the largest alluvial plains in Europe [76]. It derives from various overlapping processes and also tectonic movement, and this area was the foreland basin of the northern Apennines and southern Alps during the Paleogene [77].

Indeed, sedimentary basins can play a role in amplifying noise levels in the high-frequency range between 0.2 and 1 s [78]. Studying the behavior of the seismic response of this area is crucial because it can be influenced by liquefaction and other coseismic effects during earthquakes due to its lithological characteristics [79].

As in the case of the Po plain, the distribution of background seismic noise in the other areas of Italy in the different period intervals can be correlated both to the different distribution of the noise sources and/or also to the local geomorphological and lithological conditions that may have an effect on the amplitude and spectral characteristics of the noise present in the area [80].

On the right side of Figure 15, another possible application of noise level monitoring is shown, in this case applied to active volcanic areas. In particular, two examples are presented: at the top right, the area of the Etna volcano is shown, and at the bottom, the area of the Vesuvio volcano is shown. Volcanic eruptions can produce a variety of effects, including earthquakes, pyroclastic flows and waves, lava flows, ash and pumice fall, floods or mudflows, gas releases, and tsunamis. These two active volcanic systems cause damage in relation to their eruptions, as they have in the past in the case of Vesuvio, and in the present in the case of Etna.

In recent years, ambient seismic noise has been widely used as a means of monitoring active volcanic areas [81,82,83,84]. In fact, the monitoring of the temporal variation in the PSD level of the seismic background noise in volcano areas can be a means to study their level of activities.

Vila et al. [85] used the PSD level in the near-real-time monitoring applications of several active volcanoes, including Villarrica volcano (Chile), Tungurahua volcano (Ecuador), and Teide volcano (Spain). Rakhman et al. [86] observed how the temporal variation in the PSD level for different frequencies can describe the intensity of the event prior of the eruption. Therefore, continuous noise level monitoring can be a useful tool for understanding noise variation in terms of both intensity level and frequency contribution over time. This can potentially be useful for studying the dynamics of active volcanic systems, facilitating a deeper understanding and more comprehensive analysis of their active processes.

## 7. Conclusions

The SEISMONOISY application presented in this paper allows for the quasi-real time monitoring of noise levels of an entire seismic network, in our case applied on a national scale. Specifically, we derived a noise level index (SNLI) to quantitatively assess noise levels against Peterson’s NLNM and NHNM curves. The SNLI calculated over six period intervals ranging from 0.1 to 120 s is based on the analysis of the Probability Density Function (PDF) calculated over a total of 12 h of seismic data recorded from individual seismic stations.

As a result, seismic signals are monitored throughout the network on a 12 h cycle. The start and end times of the monitoring window were strategically chosen to separate the contributions of seismic noise during daytime and nighttime hours.

The distribution and characteristics of the environmental noise obtained with our system are compatible with the results obtained by other works that analyzed the seismic noise of the stations belonging to the Italian National Seismic Network [45,46]. These studies based their results on the analysis of seismic signals over a time period between 1 and 4 years. Our system, through the quasi-real-time monitoring approach, allows for a continuous observation of the change in the characteristics and level of the seismic noise over time in the entire monitored network, and therefore not only based on average results linked to a limited period of time.

So, the quasi-real-time noise level data and those collected over time can be used for different monitoring and performance evaluation applications both at the level of the entire seismic network and at the local level of the single seismic station.

Examples of applications at the network scale can be the evaluation of background noise for monitoring earthquakes [87] and volcanoes, the evaluation of the possible relocation of some stations and/or modifications of sensor isolation techniques, studies, and research to identify sites for new installations based on the distribution of the noise level in an area of interest, etc.

Instead, at the scale of a single station, we find the evaluation of the proper functioning of seismic stations and the possible detection of failures thanks also to the stored data for each seismic station. In conclusion, SEISMONOISY facilitates the efficient management and optimization of a seismic network, enhancing its usefulness in both practical and scientific domains.

## Figures and Tables

**Table 1:** List of seismic sensors and their distribution in the different monitored seismic networks.

Sensor Type	IV	NI	GU	MN	IY	OT	OX	ST	VR	SI	TV	Z3	3D	XE
GURALP CERTIS	2													
GURALP CMG-3ESP	1		3											
GURALP CMG-3ESP-60S				1										
GURALP CMG-3ESPC-120S	8													
GURALP CMG-3EX	8												1	
GURALP CMG-3T-120S	13													
GURALP CMG-40T-30S	5	1												
GURALP CMG-40T-60S	6													
GURALP CMG-6T-30S													1	
LENNARTZ LE3D-1S	16													
LENNARTZ LE3D-20S	5												1	
LENNARTZ LE3D-5S	36		2					1		1				
LENNARTZ LE3D-LITE	6					10		4						
LENNARTZ LE3D-lite-MkIII-1s	5													
LUNITEK TELLUS-1S	2													
LUNITEK TELLUS-5S	12													
MARK L-4C-171	2													
NANOMETRICS TRILLIUM-120	1													
NANOMETRICS TRILLIUM-120C	29				1						1	1	2	6
NANOMETRICS TRILLIUM-120S	25						5		3					
NANOMETRICS TRILLIUM-20C	1													
NANOMETRICS TRILLIUM-240S	8		2											
NANOMETRICS TRILLIUM-360GSN	1													
NANOMETRICS TRILLIUM-40S	131	1	17			2	4	2						
SARA SS08	1				5									
STRECKEISEN STS-1H-VBB				4										
STRECKEISEN STS-2-120S		3		9			2			5				
STRECKEISEN STS-2-3G				2										
TOTAL	324	5	24	16	6	12	11	7	3	6	1	1	5	6

## Data Availability

All data and developed applications presented in this article are available from the corresponding author upon request without reservation.
